# A Great Example

**Published:** 1909-01-30

**Authors:** 


### The Hospital

A JOURNAL OF

The Medical Sciences and Hospital Administration.

NEW SERIES. No. 100, VOL. IV. [No. 1172, VOL. XLV.] SATURDAY, JANUARY 30, 1909.

### A GREAT EXAMPLE.

THE London Hospital, from which our other charities might well take a lesson in the art of interesting philanthropists, has recently been the recipient of a very handsome donation from an anonymous patron, as was announced in our columns last week. The sum of £20,000 has been placed in the hands of the trustees of the medical school to be invested to the best advantage, while the income accruing therefrom is to be expended in the advancement of medical research and the promotion of higher education in medicine. This money is to be spent on increased facilities for research, not for the routine teaching of candidates for examination. Moreover, the benefits of the endowment are not to be confined to students educated at the London Hospital, but will be open to qualified medical men from any part of the British Empire, provided that they work within the walls of the London Hospital or its College.

We have often pointed out how wasteful of our best intelligences is the prevailing state of affairs in our London schools of medicine with regard to the question of research. It must be premised that no man becomes fitted for this purpose until he has served a long apprenticeship, and the necessary apprenticeship becomes daily longer. For the further we proceed into the undiscovered territories of knowledge the greater is the ground to be traversed by those who aspire to continue the exploration before ever they can commence to be real contributors to the advance. Moreover, the processes of science become progressively more complex, the result being that even the paths already charted, to continue the metaphor, are not easily trodden by those who come after, but have to be followed step by step and in the light of knowledge. It is apparent, then, that those are best fitted to advance research who have most thoroughly mastered the avenues already opened up. But under existing circumstances two things militate fatally against the best and most economical expenditure of the forces at our command. *Ex hypothesi* our researchers should be those who have had long experience—our professors and lecturers upon physiology, pathology, bacteriology. Yet as things are these very men have acquired their experience in the teeth of circumstances, one might say. The natural aptitude which in the first instance dictated their choice of a life's work has been throughout trammelled and confined by the curse of penury. Although fitted by natural endowment for the task of enlarging knowledge through original observation, their days are largely spent in giving elementary instruction and in performing routine examinations; for man must live, in spite of what has been said to the contrary, and these things are more materially profitable, for the moment at least, than is the painful and often disappointing attack upon new problems. So time passes in work which becomes almost mechanical; originality is blunted, dissipated, and often enough destroyed, by the daily round of routine drudgery which is necessary for the purse's sake.

It is certain then that what is most required is the endowment of research professorships at our large medical schools, endowments sufficient to attract the profoundest intellects of the day, appointments so ordered that the holder may devote himself with a single mind to the complex problems which await solution, instead of being forced, as he is at present, to conduct his investigations in fitful moments snatched from the formal duties which alone contribute to his exchequer. Thus only can we cease to break walnuts with Nasmyth hammers in our robust and foolish British way, and at last employ those powerful implements for the larger purposes for which they are designed and fitted. Every great hospital school commands a supply of material for research whose potentialities are endless, yet we squander it daily because our *personnel* is starved. It is galling, when one contemplates the possibilities we have at hand in London, to compare the situation here with that which obtains in America particularly, but also in some of our great provincial towns. In America far-sighted millionaires have done much to justify their existence by large, and even colossal, endowments of medical schools. The same, in a less degree, is true of some of our provinces, yet London has always lagged behind. Its very bulk is a hindrance, for London belongs to everybody, and therefore to nobody; no spirit of local pride exists to prompt the special benefactions which are so urgently required. We are far from suggesting in these querulous sentences that Londoners are not generous as a whole, for we acknowledge readily and with gratitude that their charities are enormous. But their generous impulses require the stimulus of urgent and imperative need for the alleviation of suffering. The closure of a few wards of an established hospital on account of shortage of pence may be counted upon almost with certainty to put the Londoner's hand in his pocket. But he is moved with difficulty, or not at all, when the matter involves an effort of anticipation, a projection of his mind towards future benefits to be derived from the endowment of research. The business does not stir him, or else it seems too speculative, since no honest scientist can guarantee him tangible results for his benefactions.

The work of the hospital schools in London having been so much hampered in the past, it is with the profoundest and liveliest satisfaction that we hail the gift of the anonymous patron of the London Hospital School. We do not yet know for certain how the administrators propose to apply it; whether its proceeds will be divided into a number of research scholarships such as may attract the younger generation of scientists for a year or two, or whether they will be devoted in bulk to the support of a permanent appointment to be bestowed upon some acknowledged leader of science. It seems likely that the former of these two courses will be adopted. Our personal bias is towards the latter, but in any case the benefaction is of the happiest augury. May this friend of the London Hospital find many imitators!

